# Prevalence and Risk Factors of Subclinical Tuberculosis in a Low-Incidence Setting in China

**DOI:** 10.3389/fmicb.2021.731532

**Published:** 2022-01-11

**Authors:** Peijun Tang, Ermin Liang, Xuxia Zhang, Yanjun Feng, Huafeng Song, Junchi Xu, Meiying Wu, Yu Pang

**Affiliations:** ^1^Department of Tuberculosis, The Fifth People’s Hospital of Suzhou, The Affiliated Infectious Diseases Hospital of Soochow University, Suzhou, China; ^2^Department of Bacteriology and Immunology, Beijing Tuberculosis and Thoracic Tumor Research Institute, Beijing Chest Hospital Affiliated to Capital Medical University, Beijing, China; ^3^Department of Clinical Laboratory, The Fifth People’s Hospital of Suzhou, The Affiliated Infectious Diseases Hospital of Soochow University, Suzhou, China

**Keywords:** tuberculosis, subclinical, Xpert, Beijing genotype, neutrophil-to-lymphocyte ratio

## Abstract

**Objectives:** Subclinical tuberculosis (TB) represents a substantial proportion of individuals with TB disease, although limited evidence is available to understand the epidemiological characteristics of these cases. We aimed to explore the prevalence of subclinical patients with TB and identify the underlying association between the subclinical TB cases in the study setting and the Beijing genotype.

**Methods:** A retrospective study was conducted among patients with incident TB at the Fifth People’s Hospital of Suzhou between January and December 2018. A total of 380 patients with TB were included in our analysis.

**Results:** Of the 380 patients, 81.8% were active TB cases, whereas the other 18.2% were subclinical TB cases. Compared with patients aged 65 years and older, the risk of having subclinical TB is higher among younger patients. The use of smear, culture, and Xpert identified 3, 16, and 13 subclinical TB cases, respectively. When using a combination of positive culture and Xpert results, the sensitivity improved to 33.3%. In addition, the neutrophil-to-lymphocyte ratio was significantly elevated in the active TB group compared with that in the subclinical TB group. We also observed that the proportion of the Beijing genotype in the subclinical TB group was significantly lower than that in the active TB group.

**Conclusion:** To conclude, our data demonstrate that approximately one-fifth of patients with TB were subclinical in Suzhou. *Mycobacterium tuberculosis* could be detected by the existing microbiologic diagnostics in one-third of patients with subclinical TB. The patients with subclinical TB are more prone to having low neutrophil-to-lymphocyte ratio values than those with active TB. Additionally, non-Beijing genotype strains are associated with subclinical TB.

## Introduction

Tuberculosis (TB), caused by the *Mycobacterium tuberculosis* (MTB) complex, is the leading infectious cause of mortality worldwide (Glaziou et al., 2018; World Health Organization, 2020). In 2019, approximately 10.0 million people worldwide developed active TB, and over 1.4 million people died of it (World Health Organization, 2020). Reducing the morbidity and mortality of TB requires a comprehensive understanding of its clinical pathogenic spectrum from infection to disease onset (Lenaerts et al., 2015; Drain et al., 2018). The conventional dichotomy of latent *versus* active TB has been used to describe a spectrum of infection (Bajema et al., 2019). Recently, others have clearly confirmed that a continuous spectrum of bacterial metabolic activity and antagonistic immunological responses occur in human TB infection, from latent infection to active disease (Drain et al., 2018; Kendall et al., 2021). Considering that the clinical symptoms correlate with the increasing bacillary burden and subsequent host damage (Lenaerts et al., 2015), the preceding asymptomatic periods can be defined between latent infection and active TB disease, including incipient and subclinical TB (Drain et al., 2018). The patients with viable MTB that can be detected with existing radiologic or microbiologic assays are classified as subclinical TB disease (Drain et al., 2018). Noteworthily, it is increasingly recognized that individuals with subclinical TB play an important role in the transmission of pulmonary TB (Xu Y. et al., 2019). Therefore, the World Health Organization’s (WHO’s) End TB Strategy cannot be achieved without enhanced efforts to hinder the transmission spread by the millions of people who have subclinical TB (Kendall et al., 2021).

Despite great achievements over the past decades, China had a high burden of TB with an estimated national incidence rate of 58/100,000 population in 2019 (Wang et al., 2014; World Health Organization, 2020). A population-based TB prevalence survey shows that 26.3% of the prevalent culture-positive diseases are asymptomatic (Wang et al., 2014). In view of the unsatisfactory detection limit by mycobacterial culture, the low bacillary loads of viable tubercle bacilli in subclinical TB may result in an underestimation of the burden of this asymptomatic population (Dowdy et al., 2013). However, evidence regarding the prevalence of patients with subclinical TB and their clinical characteristics remains limited in China, which poses great challenges to the formulation of effective TB control strategies.

Increasing evidence shows that the clinical MTB isolates exhibit diversity in virulence, transmissibility, and immune response they evoke (Peters et al., 2016; Orgeur and Brosch, 2018). Beijing genotype, the predominant clade of MTB in China, is considered to have a selective advantage over other clades (Pang et al., 2012). Previous clinical and epidemiological studies revealed that the emergence of the Beijing strains could be attributed to the elevated virulence of these strains, leading to rapid progression from infection to active diseases (Via et al., 2013; Ribeiro et al., 2014). Considering the increased virulence of the Beijing genotype, an interesting question is whether the patients infected with Beijing genotype strains are at low risk of subclinical TB.

To answer this question, we retrospectively collected the clinical manifestations of patients with incident TB in an urban area of Suzhou from January to December 2018. We aimed to explore the prevalence of subclinical patients with TB and identify the underlying association between the subclinical TB cases in Suzhou and Beijing genotypes.

## Patients and Methods

### Study Design

**FIGURE 1:**
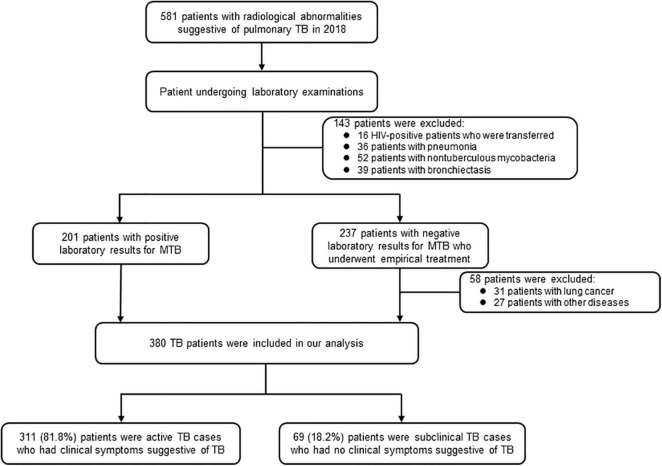
Enrolment of study participants.

We conducted a retrospective study to collect and analyze the epidemiological and clinical data of patients with incident TB at the Fifth People’s Hospital of Suzhou between January and December 2018. The hospital has 600 beds, delivering specialized treatment services to patients with TB and other infectious diseases (Xu P. et al., 2019). It provides tertiary care for patients with TB from an urban area of Suzhou and patients with severe TB from a neighboring rural area. In addition, all individuals with abnormal radiological and/or clinical symptoms suggestive of TB are referred to the hospital to complete a differential diagnosis of TB and other diseases. Routinely, patients presented with abnormal radiological features and/or clinical symptoms indicating pulmonary TB, such as cough, fever, night sweats, hemoptysis, weight loss, and chest pain, are asked to provide sputum and peripheral blood samples for laboratory testing. Patients presented with radiological abnormalities are examined with direct fluorescence microscopy, mycobacterial culture, and Xpert MTB/RIF assay. Patients with any positive microbiologic result are diagnosed with TB. Experimental therapy is initiated for patients with no microbiologic results and exclusion of other diseases. Those with an appropriate response to anti-TB treatment are also diagnosed as patients with TB (Figure 1).

We obtained the demographic and clinical data from electronic patient records to conduct comparisons between the cases of active TB and subclinical TB. The Ethics Committee of the Fifth People’s Hospital of Suzhou approved this study. A waiver of informed consent was obtained, as the data were de-identified.

### Definitions

Active TB disease was defined as a person presenting clinical symptoms, plus radiological abnormalities and/or microbiologic evidence consistent with active TB disease. Subclinical TB disease was defined as a person without any TB-associated clinical symptoms but presenting abnormalities that could be detected using radiologic and/or microbiologic assays (Drain et al., 2018).

### Laboratory Methods

Sputum samples are routinely tested to diagnose pulmonary TB. The testing methods include microscopy examination, mycobacterial culture, and Xpert. Drug susceptibility testing for first- and second-line drugs was performed for positive cultures using the proportion method as previously reported (Pang et al., 2012). In addition, QuantiFERON (QFT) IGRA (Qiagen, Venlo, Limburg, Netherlands) was offered as an immunological test for auxiliary diagnosis of TB. The hematological analysis was carried out within 30 min since the arrival of fresh whole blood. Complete blood counts were measured using a Sysmex XN-1000 automated cell counter (Sysmex, Kobe, Japan) and platelets measured in units × 10^9^/L. Lymphocyte subset percentages were analyzed using a FACSCanto flow cytometer (BD, Franklin Lakes, NJ, United States).

### Spoligotyping

The MTB isolates of the included patients were subcultured on Löwenstein–Jensen medium for 4 weeks. The crude genomic DNA was extracted from fresh bacterial colonies with a simple boiling method as previously reported (Huo et al., 2020), which was used as the template DNA for subsequent genotyping. Spoligotyping was conducted using a commercial kit on the basis of multicolor melting curve analysis (Zeesan Biotech, China) (Zeng et al., 2018). Forty-three fluorescent probes were used to detect the 43 spacers of the direct repeat region, and the preinstalled analysis software automatically yielded the results in a binary format. The original data were submitted to the SpolDB 4.0 database^1^ to identify the spoligotype international type of each isolate.

### Statistical Analysis

All laboratory results were presented as mean ± standard deviation. The unpaired student *t-test* was used to analyze normally distributed continuous data, whereas the Mann–Whitney *U*-test was used to compare non-normally distributed data. The paired *t-*test was used when laboratory data were in the form of matched pairs. The Pearson chi-square test assessed the comparison of categorical variables. Univariate analyses were performed on multiple demographic and clinical variables to analyze risk factors for subclinical TB cases first. We included the variables that were statistically significant in univariate analysis and important covariates potentially affecting the distribution of subclinical TB cases into the multivariate model. All statistical analysis was performed using the SPSS program (SPSS version 17.0, SPSS Inc., Chicago, IL, United States). Statistical significance was accepted at a 95% level.

## Results

### Patients

A total of 581 patients with indicators suggestive of pulmonary TB sought healthcare at the Fifth People’s Hospital of Suzhou during the study period. We excluded 201 cases from the sample due to diagnosis with non-TB diseases or being human immunodeficiency virus (HIV)-positive, leaving 380 TB cases in our final analysis. Of the 380 patients, 311 (81.8%) were active TB cases, and the other 69 (18.2%) were subclinical TB cases (Figure 1).

We summarized the demographic and clinical characteristics of the patients with subclinical TB compared with patients with active TB in Table 1. The distribution of subclinical TB differed among age groups. Using patients < 25 years of age as a control group, we found that older people (≥65 years of age) were less prone to have subclinical TB [adjusted odds ratio (aOR): 0.280, 95% confidence interval (CI): 0.103–0.766], and patients had a lower risk of subclinical TB with increasing age (aOR: 0.972, 95% CI: 0.494–1.914 for patients 25–44 years of age; aOR: 0.528, 95% CI: 0.223–1.251 for patients 45–64 years of age). In addition, patients with subclinical TB had a lower risk for multiple lung lesions than patients with active TB (aOR: 0.248, 95% CI: 0.141–0.434).

**TABLE 1:** Demographic and clinical characteristics of patients enrolled in this study.

Characteristics	No. of patients (%)			Crude OR (95% CI)	*P*-value	Adjusted OR (95% CI)	*P*-value
	Subclinical TB cases (*N* = 69)	Active TB cases (*N* = 311)	Total (*N* = 380)				
**Age (years)**							
< 25	21 (30.4)	58 (18.6)	79 (20.8)	Ref.		Ref.	
25–44	31 (44.9)	101 (32.5)	132 (34.7)	0.848 (0.446–1.610)	0.614	0.972 (0.494–1.914)	0.934
45–64	11 (15.9)	73 (23.5)	84 (22.1)	0.416 (0.186–0.933)	0.033	0.528 (0.223–1.251)	0.147
≥ 65	6 (8.7)	79 (25.4)	85 (22.4)	0.210 (0.080–0.553)	0.002	0.280 (0.103–0.766)	0.013
**Sex**							
Male	52 (75.4)	232 (74.6)	284 (74.7)	Ref.		Ref.	
Female	17 (24.6)	79 (25.4)	96 (25.3)	0.960 (0.525–1.757)	0.895	0.897 (0.470–1.713)	0.743
**Residence**							
Rural	37 (53.6)	176 (56.6)	213 (56.1)	Ref.		Ref.	
Urban	32 (46.4)	135 (43.4)	167 (43.9)	1.128 (0.668–1.903)	0.653	1.048 (0.597–1.839)	0.869
**Lesions in pulmonary**							
Single	37 (53.6)	63 (20.3)	100 (26.3)	Ref.		Ref.	
Multiple	32 (46.4)	248 (79.7)	280 (73.7)	0.220 (0.127–0.380)	<0.001	0.248 (0.141–0.434)	< 0.001
**Cavitation**							
No	59 (85.5)	266 (85.5)	325 (85.5)	Ref.		Ref.	
Yes	10 (14.5)	45 (14.5)	55 (14.5)	1.002 (0.478–2.102)	0.996	1.126 (0.508–2.497)	0.770
**Comorbidity**							
No	64 (92.8)	279 (89.7)	343 (90.3)	Ref.		Ref.	
Yes	5 (7.2)	32 (10.3)	37 (9.7)	0.681 (0.255–1.817)	0.443	0.875 (0.303–2.531)	0.806

### Laboratory Results of Subclinical vs. Active Tuberculosis Cases

**FIGURE 2:**
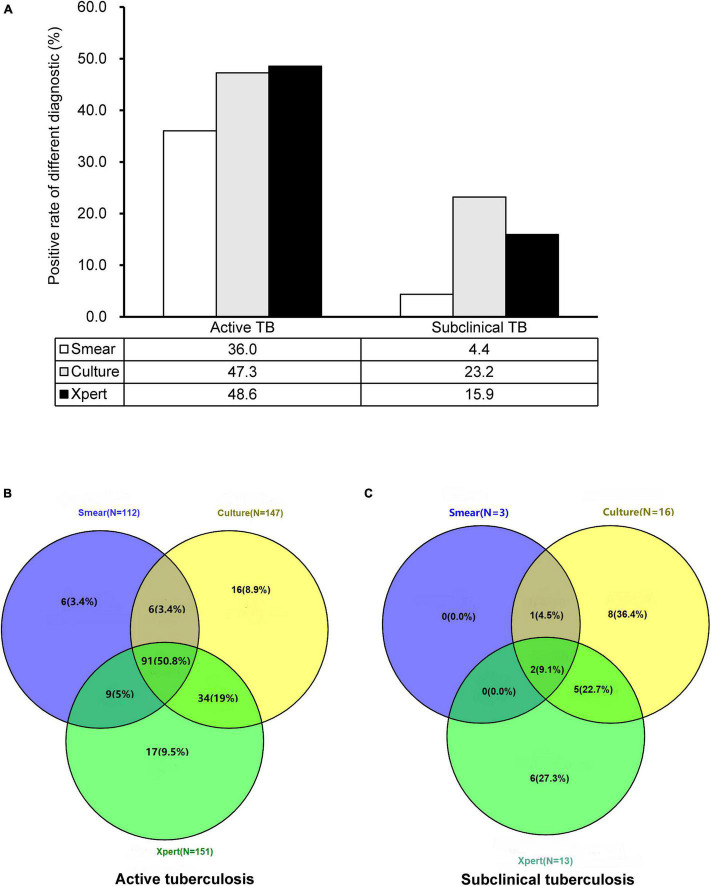
Comparison of smear, mycobacterial culture, and Xpert for diagnosis of active and subclinical TB cases. **(A)** Positive rate of different diagnostics for diagnosing TB cases. **(B)** Concordance of smear, culture, and Xpert results on active TB cases. **(C)** Concordance of smear, culture, and Xpert results on subclinical TB cases.

Overall, 22 (31.9%) of 69 subclinical TB cases had smear, culture, and/or Xpert-positive evidence of MTB, which was significantly lower than that in the active TB cases (179/311, 57.6%, *P* < 0.001). The use of smear, culture, and Xpert identified 3 (4.3%), 16 (23.2%), and 13 (18.8%) subclinical TB cases, respectively. When a positive culture and Xpert result were combined, the sensitivity improved to 33.3% (23/69). However, additional smear microscopy could not provide any value for the diagnosis of these cases. In addition, low bacterial loads, as determined by mycobacterial growth indicator tube time to positivity, were observed in the subclinical TB group compared with the active TB group (13.8 days for active TB group *versus* 29.6 days). A similar relationship was seen using Xpert quantification as a marker of bacterial load. Of 13 cases yielding positive Xpert results, 12 (92.3%) had a very low bacterial load, and the remaining one (7.7%) had a low bacterial load. The proportion of cases with very low bacterial load in the subclinical TB group was significantly higher than that in the active TB group (*P* < 0.05) (Figure 2).

We further analyzed the results of blood cell count between subclinical TB and active TB cases. As shown in Table 2, subclinical TB cases were more likely to have a high proportion of lymphocytes (31.23 ± 7.99) but a low proportion of neutrophils (57.64 ± 8.96) than active TB cases (23.72 ± 11.29 for lymphocyte and 63.99 ± 12.58 for neutrophil). Correspondingly, the neutrophil-to-lymphocyte ratio (NLR) was significantly elevated in the active TB cohort compared with that in the subclinical TB cohort. In addition, the value of hemoglobin in subclinical TB cases was significantly higher than those in active TB cases.

**TABLE 2:** Comparison of laboratory results between subclinical and active TB cases.

Characteristics^a^	Subclinical TB cases (Mean ± SD, *N* = 69)	Active TB cases (Mean ± SD, *n* = 311)	*P-*value
T lymphocytes (%)	67.93 ± 7.19	67.28 ± 9.76	0.555
CD4+ (%)	37.91 ± 7.74	36.49 ± 8.56	0.232
CD8+ (%)	27.92 ± 7.23	28.56 ± 9.2	0.608
CD4+/CD8+	1.52 ± 0.76	1.58 ± 1.89	0.830
B lymphocytes (%)	14.78 ± 4.73	12.23 ± 5.52	0.001
NK cells (%)	14.47 ± 5.86	17.66 ± 9.3	0.001
Leucocytes (× 10^9^/L)	5.91 ± 1.46	6.55 ± 2.39	0.005
Neutrophils (× 10^9^/L)	3.45 ± 1.19	4.93 ± 10.3	0.236
Neutrophils (%)	57.64 ± 8.96	63.99 ± 12.58	< 0.001
Lymphocytes (× 10^9^/L)	1.8 ± 0.54	1.52 ± 1.77	0.195
Lymphocytes (%)	31.23 ± 7.99	23.72 ± 11.29	< 0.001
NLR	2.08 ± 1.03	4.83 ± 14.56	< 0.001
Hemoglobin (g/L)	144.48 ± 18.23	130.08 ± 20.82	< 0.001
Platelets (× 10^9^/L)	232.26 ± 56.98	258.54 ± 94.47	0.003

*^a^SD, standard deviation; NK, natural killer; NLR, neutrophil-to-lymphocyte ratio.*

### Association Between Beijing Genotype and Subclinical Tuberculosis

**FIGURE 3:**
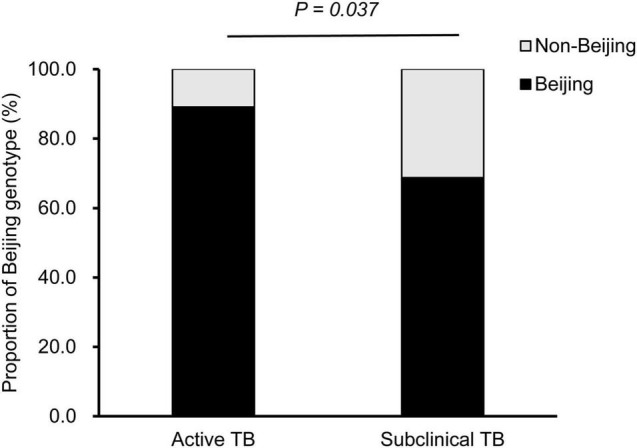
Distribution of Beijing and non-Beijing genotypes between active and subclinical TB cases.

To determine the association between Beijing genotype and subclinical TB, the positive cultures from patients were genotyped with the commercial Mcspoligotyping method. Of the 163 MTB isolates, 142 (87.1%) were classified as Beijing genotypes, and the other 21 (12.9%) were non-Beijing genotypes. In the active TB group, 131 of 147 MTB isolates were identified as Beijing genotypes, whereas only 68.75% of isolates in the subclinical TB group were Beijing genotypes. Statistical analysis revealed a significant difference in the proportion of Beijing genotype between subclinical and active TB cases (*P* = 0.037) (Figure 3).

## Discussion

Subclinical TB cases are gaining increasing attention due to their high potential to exacerbate TB transmission (Oni et al., 2011; Kendall et al., 2021). Estimates of their contribution to the transmission of infectious tubercle bacilli are hampered by the limited evidence available. This study is the first one that describes the prevalence of subclinical TB among all TB cases in a low-incidence setting in China. Our data demonstrated that approximately one-fifth of patients with TB were subclinical in the study region. Based on the review of 12 national prevalence surveys in Asia, the percentages of all bacteriologically confirmed TB cases who did not report TB symptoms ranged from 40% in Pakistan to 79% in Myanmar (Onozaki et al., 2015; Drain et al., 2018). In line with our observation, nearly one-quarter of TB cases were subclinical TB among HIV-infected adults in a South Africa cohort (Bajema et al., 2019). Notably, diagnosis of TB in the previous population-based TB surveys often required symptoms of cough lasting 2 or more weeks (Onozaki et al., 2015). Hence, these discrepancies in definitions of TB-associated symptoms, as well as epidemiological settings and screening tools, may be plausible explanations for the different prevalence of subclinical TB across studies.

A growing body of literature indicates that individuals without recognizable symptoms are capable of producing infectious droplets to potentially accumulate community transmission of infectious pathogens (Bousema et al., 2014; Bai et al., 2020). Our findings support the hypothesis that subclinical TB is responsible for a fraction of MTB transmission, given that tubercle bacilli could be detected by the existing microbiologic diagnostics in one-third of individuals with subclinical TB. On the one hand, a majority of subclinical TB cases yielded negative laboratory results, indicating low sputum bacillary loads in their sputum. This poor detection rate highlights the urgent need for highly sensitive diagnostic assays such as Xpert MTB/RIF Ultra to facilitate the early identification of subclinical TB (Opota et al., 2019). On the other hand, the transmissibility of MTB can be considered to be a function of the degree of infectivity, the duration of exposure, and the availability of susceptible contacts (Drain et al., 2018). Although a significant low bacillary burden is observed among the subclinical TB cases, they can potentially cause a substantial fraction of transmission on a population level due to its high prevalence and long duration. Given that a substantial MTB transmission originates from people with unrecognizable symptoms, it is essential to adopt better active diagnosis strategies for greater progress toward the WHO’s ambitious End TB Strategy.

A key question is an extent to which individuals at high risk for subclinical TB should be prioritized for testing and treatment. Several studies have reported that groups at high risk for subclinical TB are similar to those for active TB, including residence in high-incidence settings and persons with a history of TB (Oni et al., 2011; van’t Hoog et al., 2011; Drain et al., 2018). In this cohort, we found that the prevalence of subclinical TB substantially decreases with aging, whereas the reverse is true for active TB. The maintenance of subclinical TB indicates the heterogeneity in the time from initial infection to the development of active TB. The rapid development may be associated with the weakened immunity that fails to control the actively replicating bacilli. Therefore, these corresponding trends may be explained by changes during aging (Licastro et al., 2005; Nikolich-Zugich, 2018), thus triggering clinical or bacteriological progression. Similarly, a recent modeling study where HIV-positive people comprise a small proportion of subclinical TB cases conducted by Kendall and colleagues showed that HIV-positive individuals progress more rapidly from infection to disease onset (Kendall et al., 2021). Taken together, our data suggest that the proportion of subclinical TB is conversely affected by the immunity status of individuals.

Recent studies have confirmed that the NLR has been associated with the severity or prognosis of several infectious diseases, such as coronavirus disease 2019 and community-acquired pneumonia (de Jager et al., 2012; Ciccullo et al., 2020). A study from Thai by Miyahara et al. (2019) demonstrated that the high NLR increased the risk of TB in HIV-infected individuals. In the present study, we found that the individuals with subclinical TB were more prone to having low NLR values than those with active TB. During the natural history of TB, lymphocytes are the most predominant immune cells against TB infection (Jones et al., 1997; Panteleev et al., 2017), whereas neutrophils participate in granuloma formation and/or pulmonary destruction (Ong et al., 2015; Panteleev et al., 2017). As a consequence, the elevated NLR may be used as an indicator for attenuated ability to inhibit *in vivo* multiplication of tubercle bacilli.

Another interesting finding in our study was a higher proportion of non-Beijing genotype strains in the subclinical TB group. Numerous experimental studies have documented that MTB strains show substantial variation in their virulence and immunogenicity between different phylogenetic lineages (Lopez et al., 2003; Reiling et al., 2013). Of these lineages, Beijing genotype strains exhibit higher virulence and more rapid progression to disease onset in animal models and have been manifested in several human TB outbreaks from a public health perspective (Narvskaya et al., 2002; Hou et al., 2020). The predominant Beijing genotypes in China may facilitate the progression of TB, thus potentially lowering the prevalence of subclinical TB among the individuals affected by MTB in our Chinese cohort.

There are several limitations to this study. First, this study has a very small sample size and only collects data from one hospital, which may weaken the robustness of our conclusion. Second, a majority of residents in Suzhou had routine health examinations annually; however, the partial exclusion of participants may underscore the burden of subclinical TB in this region. Third, only chest radiography or computerized tomography were used for screening patients with abnormal radiological features rather than positron emission tomography, which may result in the underestimation of subclinical TB cases with subtle signs at an early stage. Finally, HIV-infected individuals were at low risk for the development of subclinical TB. Due to the low prevalence of HIV incidence, we could not validate it in our cohort. Despite these limitations, this study is the first one that provides a snapshot of subclinical TB burden in a low-incidence setting in China.

To conclude, our data demonstrate that approximately one-fifth of patients with TB are subclinical in Suzhou. Tubercle bacilli could be detected by the existing microbiologic diagnostics in one-third of individuals with subclinical TB. The prevalence of subclinical TB substantially decreases with aging. Individuals with subclinical TB are more prone to having low NRL values than those with active TB. In addition, a higher proportion of non-Beijing genotype strains is observed in the subclinical TB group. Given that a substantial MTB transmission originates from people with unrecognizable symptoms, better active diagnosis strategies are necessary to facilitate faster progress toward the WHO’s ambitious End TB Strategy.

## Data Availability Statement

The original contributions presented in the study are included in the article/supplementary material, further inquiries can be directed to the corresponding authors.

## Ethics Statement

The studies involving human participants were reviewed and approved by the Ethics Committee of the Fifth People’s Hospital of Suzhou. The patients/participants provided their written informed consent to participate in this study. Written informed consent was obtained from the individual(s) for the publication of any potentially identifiable images or data included in this article.

## Author Contributions

YP and MW designed and supervised the study and revised the manuscript. PT, EL, YF, and JX collected clinical information and samples. XZ and HS conducted laboratory tests. PT and EL analyzed and interpreted the data. PT, EL, XZ, and YF wrote the manuscript. All authors meet the requirements for authorship and critically reviewed and approved the final manuscript.

## Conflict of Interest

The authors declare that the research was conducted in the absence of any commercial or financial relationships that could be construed as a potential conflict of interest.

## Publisher’s Note

All claims expressed in this article are solely those of the authors and do not necessarily represent those of their affiliated organizations, or those of the publisher, the editors and the reviewers. Any product that may be evaluated in this article, or claim that may be made by its manufacturer, is not guaranteed or endorsed by the publisher.

## Footnotes

1
http://www.pasteur-guadeloupe.fr:8081/SITVIT_ONLINE/query
